# Eco-friendly, compact, and cost-efficient triboelectric nanogenerator for renewable energy harvesting and smart motion sensing

**DOI:** 10.1016/j.heliyon.2024.e28482

**Published:** 2024-03-25

**Authors:** Enrique Delgado-Alvarado, Jaime Martínez-Castillo, Enrique A. Morales-González, José Amir González-Calderón, Edgar F. Armendáriz- Alonso, Gustavo M. Rodríguez-Liñán, Ricardo López-Esparza, José Hernández-Hernández, Ernesto A. Elvira-Hernández, Agustín L. Herrera-May

**Affiliations:** a Micro and Nanotechnology Research Center, Universidad Veracruzana, Boca del Río, 94294, Veracruz, Mexico; b Cátedras CONAHCYT-Instituto de Física, Universidad Autónoma de San Luis Potosí, San Luis Potosí, 78290, San Luis Potosí, Mexico; c Doctorado Institucional en Ingenieria y Ciencia de Materiales, Universidad Autónoma de San Luis Potosí, 78210, San Luis Potosí, Mexico; d Investigadores por Mexico, Centro de Geociencias, Universidad Nacional Autónoma de Mexico, Juriquilla, 76230, Querétaro, Mexico; e Departamento de Física, Universidad de Sonora, Hermosillo, 83000, Sonora, Mexico; f Facultad de Ingeniería Mecánica y Ciencias Navales, Universidad Veracruzana, Boca del Río, 94294, Veracruz, Mexico; g Maestría en Ingeniería Aplicada, Facultad de Ingeniería de la Construcción y el Hábitat, Universidad Veracruzana, Boca del Río, 94294, Veracruz, Mexico

**Keywords:** Eco-friendly materials, Energy harvesting, Green energy, Nopal powder, Sustainable energy, Triboelectric nanogenerator

## Abstract

In recent years, the growth of Internet of Things devices has increased the use of sustainable energy sources. An alternative technology is offered by triboelectric nanogenerators (TENGs) that can harvest green energy and convert it into electrical energy. Herein, we assessed three different nopal powder types that were used as triboelectric layers of eco-friendly and sustainable TENGs for renewable energy harvesting from environmental vibrations and powering electronic devices. These nanogenerators were fabricated using waste and recycled materials with a compact design for easy transportation and collocation on non-homogeneous surfaces of different vibration or motion sources. In addition, these TENGs have advantages such as high output performance, stable output voltage, lightweight, low-cost materials, and a simple fabrication process. These nanogenerators use the contact-separation mode between two triboelectric layers to convert the vibration energy into electrical energy. TENG with the best output performance is based on dehydrated nopal powder, generating an output power density of 2.145 mWm^−2^ with a load resistance of 39.97 MΩ under 3g acceleration and 25 Hz operating frequency. The proposed TENGs have stable output voltages during 22500 operating cycles. These nanogenerators can light 116 ultra-bright blue commercial LEDs and power a digital calculator. Also, the TENGs can be used as a chess clock connected to a mobile phone app for smart motion sensing. These nanogenerators can harvest renewable vibration energy and power electronic devices, sensors, and smart motion sensing.

## Highlights

• The TENG only uses waste and recycled materials for green energy harvesting.
• The TENG can be used on non-homogeneous surfaces of different vibration sources.
• The TENG can be applied for smart motion sensing and powering electronic devices.

## Graphical abstract

**Figure:**
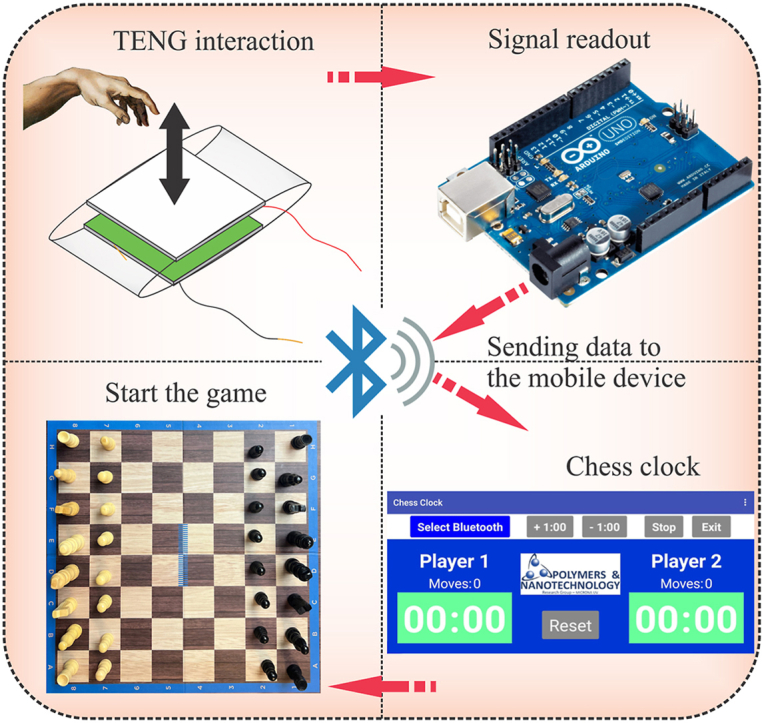

## 1 Introduction

The rapid growth of electronic devices and wireless sensor networks connected to the Internet of Things (IoT) will transform health monitoring, medical treatment, communication, entertainment, transportation, environmental protection, infrastructure monitoring, and security [[1], [2], [3], [4]]. However, these devices require sustainable energy sources from the environment [[5], [6], [7], [8], [9]]. In addition, these energy sources can be harvested from vibration, wind, thermal, raindrop, and human motion [[10], [11], [12], [13]]. To harvest these green energy sources, the triboelectric nanogenerator (TENG) is a recent technology that includes advantages such as high electrical output performance, simple structural designs, and lightweight and diverse triboelectric layers [[14], [15], [16]]. Thus, the TENGs could replace conventional batteries to satisfy the new demand for powering IoT devices [17]. These conventional batteries have limited service life and contain harmful substances that can pollute the environment [18,19]. The TENGs need diode rectifier bridges to convert their variable output voltage to direct current (DC) output voltage. This DC output voltage can be stored in supercapacitors with more electrical charge and discharge cycles than conventional batteries.

The TENG output performance depends on triboelectrification contact and electrostatic induction between triboelectric materials [20,21]. Thus, this performance can be modified due to the surface charge density, contact area, thickness, and gap of the triboelectric layers and the operating frequency and force applied to the triboelectric materials [[22], [23], [24]].

Recent researchers [[25], [26], [27], [28], [29]] have developed TENGs by integrating triboelectric layers with natural and manufactured polymers. The TENGs based on natural polymers have an eco-friendly and non-complex manufacturing process, easy degradation, and are renewable, contributing to the circular economy and reducing production costs. For instance, Jiao et al. [30] fabricated a sandwich structured TENG based on wheat bread and celery cabbage, which can achieve a maximum power density of 1.0 mWm^−2^, maximum voltage, and current close to 15 V and 3 μA, respectively. This TENG employs bio-degradable natural materials for green energy harvesting with applications into commercial LEDs and alarm dispositive. Due to degradable and porous natural materials with low mechanical strength that used this TENG, its output performance could have large variations. Thus, this nanogenerator requires more experimental tests to study its stability and reliability. On the other hand, Khan et al. [31] developed low-cost biocompatible TENG composed of a tribopositive layer of chicken skin (CS). This nanogenerator generates an open-circuit voltage and short-circuit current of 123 V and 20 μA, as well as a power density of 2000 mWm^−2^ at 20 MΩ of load resistance. This CS-TENG has stable performance with more than 52000 cycles and can harvest energy to light commercial LEDs, power a calculator, and detect motions of the human body. The performance of this CS-TENG can be altered by the thickness, surface area, and operating frequency of the CS-triboelectric layer, as well as its wear due to friction with the triboelectric layer of Kapton. Another TENG with tribopositive layer of lignocellulosic waste fruit shell was reported by Saqid et al. [32]. They tested the performance of their nanogenerator using three different tribopositive materials such as almond, walnut, and pistachio. For the case of pistachio triboelectric layer, the TENG reached a maximum power density, open-circuit voltage, and short circuit current of 4161.4 mWm^−2^, 700 V, and 95 μA, respectively. This eco-friendly and sustainable nanogenerator was used to power commercial LEDs, stopwatch, and electronic calculator. Nevertheless, the open-circuit voltage and short-circuit current of this TENG registered a non-uniform behavior. Furthermore, the environmental humidity can significantly affect the output performance of this nanogenerator. Also, more research on the wear of the triboelectric layers under different operating frequencies and pressures can help to study the performance of these types of nanogenerators. Babu et al. [33] studied the performance of another eco-friendly TENG with a triboelectric layer fabricated with leaf powder of the Rumex *vesicarius* plant. This nanogenerator registered a maximum power density, open-circuit voltage, and short-circuit current of 1.894 mWm^−2^, 3.86 V, and 3.78 μA, respectively. However, the high roughness of the leaf powder film caused less contact surface with the other PET triboelectric layer, decreasing the output performance of this nanogenerator. In addition, the output performance of this TENG registered variations during its operation. Also, this TENG used a weak structural frame based on two cardboard sheets that can be damaged by mechanical impacts and environmental humidity. Shaukat et al. [34] designed a sustainable TENG based on bio-waste sunflower husks powder as a triboelectric layer. This nanogenerator had a power density, open-circuit voltage, and short-circuit current of 480 mWm^−2^, 488 V, and 28.5 μA, respectively. This cost-effective and eco-friendly nanogenerator can harvest biomechanical energy to convert it into electrical energy, powering commercial LEDs, calculator, and stopwatch. However, this TENG had non-uniform output performance under different separation distances between the triboelectric layers. The output voltage of this nanogenerator significantly decreases when the environmental humidity increases.

The nopal (*opuntia ficus-indica*) is a typical cactus of semi-arid and arid environments that grows in Latin America, South Africa, and Mediterranean countries. Herein, we propose three eco-friendly and sustainable TENGs based on three different nopal powders: (M1) fresh nopal powder, (M2) dehydrated nopal powder, and (M3) old nopal powder. The different nopal powders act as triboelectric layers, which were deposited to copper-electrode film adhered to a bakelite plate of 5.22 cm × 5.22 cm. The second triboelectric layer of each TENG was a polyimide film tape adhered to another copper-electrode/bakelite plate. For each TENG, both triboelectric layers were supported using a recycled PET sheet for working into a separation-contact transduction mechanism. Thus, the TENGs can harvest environmental vibration energy and transform it into electrical energy. The three TENGs have high output performance, stable output voltage, lightweight and compact structure, a cost-effective fabrication process, as well as simple output signal processing under acceleration and frequency of 3g and 25 Hz, respectively. The best output performance of the TENG was obtained using the (M2) dehydrated nopal powder. Thus, the M2-based TENG generates an output power density of 2.145 mWm^−2^, an output voltage of 15.28 V, and a current of 0.38 μA with a load resistance of 39.97 MΩ. Even after 22500 operation cycles, this TENG has output voltage with stable behavior. In addition, the TENG was used to power a digital calculator and 116 ultra-bright blue and green commercial LEDs. Also, a chess clock connected to a mobile phone app for smart motion sensing was developed using the proposed TENG. The compact design of the TENGs can allow their easy transportation and collocation on non-homogeneous surfaces of different vibration or motion sources. Also, the design of the nanogenerators is suitable for green energy harvesting from environmental vibrations, wind, and biomechanical motion. Thus, these nanogenerators based on waste and recycled materials can enable pollution-free and sustainable energy generation systems. Arrays of these nanogenerators could be employed to light LED lamps and power IoT electronic devices. Another characteristic of the proposed TENGs is their ability to perform as self-powered sensors.

## 2 Experimental section

### 2.1 Materials

We fabricated three TENGs using three different nopal powder types as the first triboelectric layer and a second triboelectric layer of polyimide tape. The three nopal powder types are named as follows: (M1) fresh nopal powder, (M2) dehydrated nopal powder, and (M3) old nopal powder. To fabricate the M1-nopal powder sample, we obtained fresh nopal from a Veracruz City market in Mexico. First, the nopal was cleaned to remove the soil particles, areoles, and spines. In the second step, we removed and cut the outer skin of the nopal into strips of 3 cm thickness. These strips were placed on the surface of aluminum foil and dried using a 200 W white light reflector for 12 h under a relative humidity of 40 %. In the third step, the nopal was ground employing a blender (Osterizer 6640-13 model) at 8000 rpm for 5 min. Next, we employ a wooden mortar to get the nopal powder from the ground nopal. On the other hand, the M2-nopal powder was acquired from Natura Bio Foods (NBF) (Zapopan, Mexico) [35]. This nopal powder is derived from nopal-dehydrated leaves. Finally, the M3-nopal powder was obtained from M1-nopal powder that was stored in a Petri dish at room temperature of 25 °C for 4 months. Fig. S1 of supplementary material depicts the three nopal powder samples (M1, M2, and M3). Later, each nopal powder sample was adhered to on a copper-coated bakelite electrode plate (dimensions of 52.2 mm × 52.2 mm × 1.5 mm). Also, three polyimide tapes with 52.2 mm width and 60 μm thickness were adhered to three copper-coated bakelite electrode plates (dimensions of 52.2 mm × 52.2 mm × 1.5 mm). This polyimide tape (50 mm width and 60 μm thickness) was acquired from Steren (Mexico City, Mexico) [36].

### 2.2 Working mechanism

**Fig. 1:**
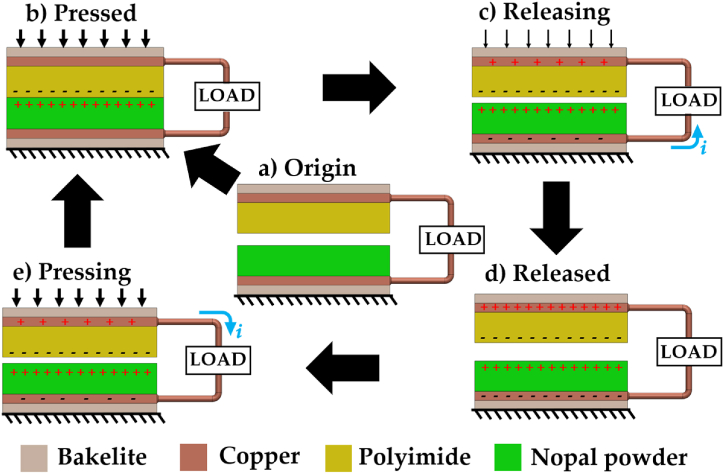
Schematic view of the working mechanism of the nopal powder-based TENG.

Fig. 1 depicts the stages of the separation-contact transduction mechanism of the proposed TENG. This mechanism combines the coupling of the contact electrification and electrostatic induction processes. Fig. 1 (a) shows the initial state of this mechanism, in which no charges are generated or induced due to no potential differences between the top and bottom electrodes. The upper and bottom plates of the TENG are composed of a copper-coated bakelite/polyimide film tape and nopal powder/cooper-coated bakelite, respectively. Next, by applying an external force on the upper plate of the nanogenerator, the two triboelectric materials achieve physical contact. In this case, electrostatic charges are accumulated on the contact surface of the TENG due to contact electrification (Fig. 1(b)). Next, the top plate moves and establishes contact with the bottom plate. Thus, the polyimide surface becomes negatively charged, while the nopal powder surface acquires a positive charge. In the following stage, the external force is removed, and the two plates are released. In this stage, the following electrical charges with opposite polarity to those of the triboelectric layers are generated on the surfaces of the top and bottom electrodes (Fig. 1(c)). This charge difference causes an electrical potential difference between the two electrodes, generating a current through the connected load resistance. The current flow is stopped when the separation distance between the two plates achieves its maximum value (Fig. 1(d)). However, reapplying an external force (by pressing) on the upper plate reduces its separation distance from the bottom plate, producing a potential difference between the electrodes and a current flow through load resistance, as shown in Fig. 1(e). Finally, periodic contact and separation between the triboelectric layers facilitate the oscillating current between both electrodes.

### 2.3 Fabrication

**Fig. 2:**
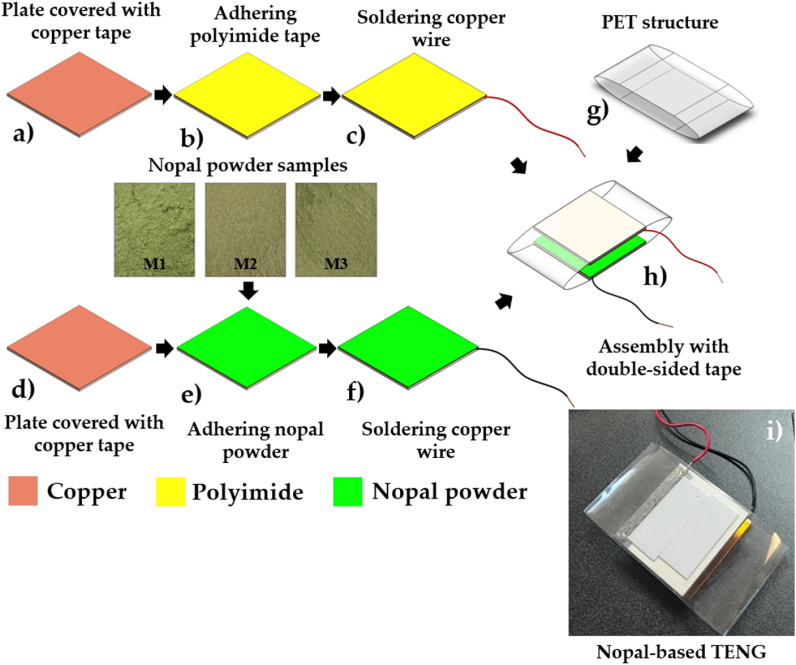
Fabrication of the nopal powder-based TENG. (a–c) Stages to develop the upper triboelectric layer with polyimide tape. (d–f) Stages to fabricate the bottom triboelectric layer with nopal powder. (g–i) Assembly of both triboelectric layers in a recycled PET sheet to obtain the nopal powder-based TENG.

In the fabrication of the nopal powder-based TENG, the outer surfaces of two bakelite plates (52.2 mm × 52.2 mm × 1.5 mm) are covered with a copper tape (62.2 mm × 52.2 mm × 66 μm) as shown in Fig. 2 (a,d). A section (10 mm × 52.2 mm × 66 μm) of the copper tape exceeds the bakelite plate, which is adhered to the underside of this plate. In addition, a polyimide film adhesive tape (52.2 mm × 52.2 mm × 60 μm) was adhered to the copper electrode located on the upper plate (Fig. 2(b)). Next, a 0.644 mm-diameter tinned copper wire was soldered to the section of the copper tape adhered underside of the plate (Fig. 2(c)). Furthermore, the nopal powder was adhered to the copper electrode placed on the lower plate (Fig. 2(e)). To adhere the nopal powder to the copper electrode/bakelite plate, a controlled layer of white adhesive was applied to the outer surface of the copper electrode using the spin-coating process at a rotation speed of 1550 rpm for 15 s. The nopal powder was evenly spread across the glue-coated surface of the copper electrode. In the following step, we collocated the nopal powder adhered to a copper tape/bakelite plate to air dry for 24 h at ambient room temperature. Subsequently, the residues of nopal powder were removed using an air compressor. After, we solder a tinned copper wire to the electrode on the lower plate (Fig. 2(f)). For the structural frame of the nanogenerator, a recycled PET sheet (106.79 mm × 52.20 mm × 0.30 mm) was used as shown in Fig. 2(g). Fig. 2(h) depicts both bakelite plates glued to a recycled PET sheet, forming the complete structure of the nopal powder-based TENG. Fig. 2(i)) illustrates the two triboelectric layers and bakelite plates glued to the PET sheet to develop the nopal powder-based TENG. In this nanogenerator, the polyimide film acts as an electronegative triboelectric layer, while nopal powder operates as the electropositive triboelectric layer. Each proposed TENG has a mass close to 25 g, considering its bakelite plates, electrodes, both triboelectric layers and PET sheet.

### 2.4 Setup

**Fig. 3:**
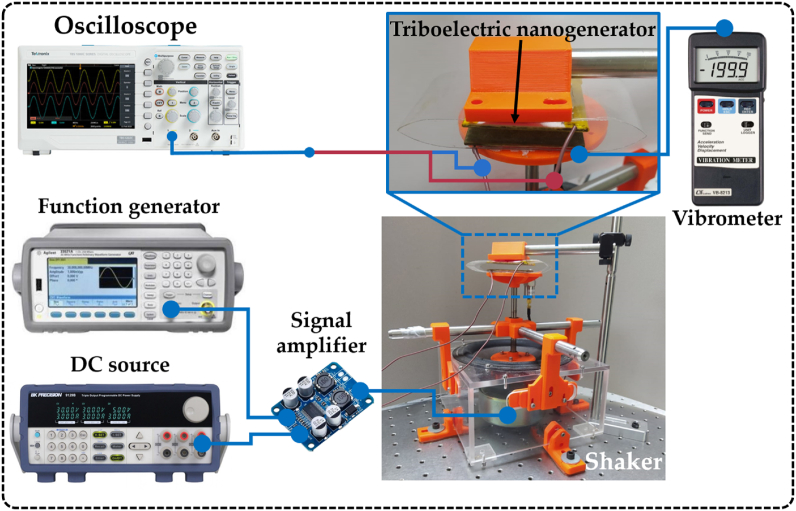
The setup to measure the output performance of the nopal powder-based TENG.

The different equipment used to measure the output performance of the TENG is shown in Fig. 3. A shaker is employed to apply controlled vibrations to the triboelectric nanogenerator. These vibrations are generated employing a function generator of Agilent 33500B series, a signal amplifier of Texas Instruments TPA3118, and a direct current source of Agilent E3631A. To measure the vibration accelerations, a LUTRON VB-8213 vibrometer was employed. Furthermore, a TEKTRONIX TBS1052C digital oscilloscope was interconnected with the two copper electrodes of the proposed nanogenerator. This setup assessed the nanogenerator's output performance under various vibration amplitudes.

## 3 Results and discussion

This section describes the characterization of the functional groups and surface (SEM) to examine the surface morphology of the triboelectric materials, as well as the output performance of the triboelectric nanogenerator. We use the attenuated total reflection (ATR) Fourier transform infrared (FTIR) spectroscopy for the nopal powder to identify the functional groups that influence its triboelectric properties.

### 3.1 FTIR

**Fig. 4:**
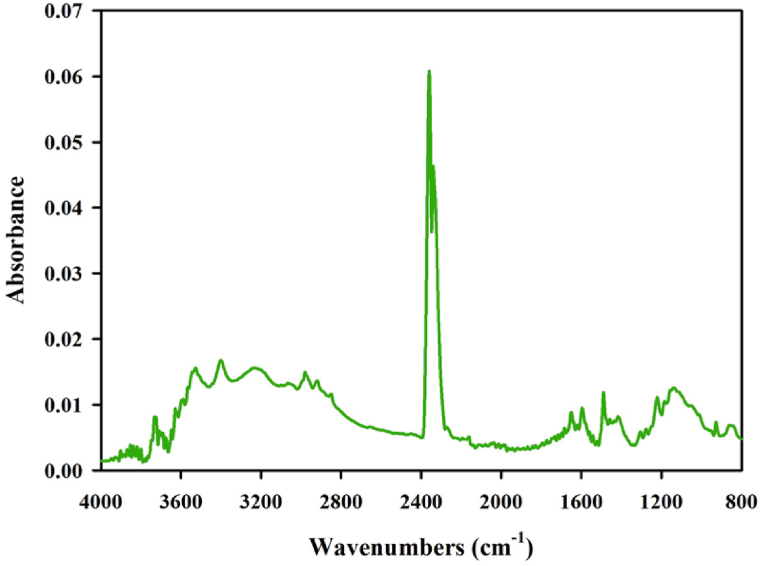
ATR-FTIR spectra from nopal powder used as triboelectric material of the proposed TENG.

Fig. 4 shows the ATR FTIR spectroscopy for nopal powder, in which the spectrum analysis between 3500 cm^−1^ and 3000 cm^−1^ indicates a bandwidth of O–H stretching vibrations. The reduction in the intensity of the OH signal at 3300 cm^−1^ observed in the nopal cellulose sample is due to the percentage of nopal powder. It suggests a correlation between signal intensity and the rate of nopal powder. Additionally, it has been previously demonstrated [37,38] that the characteristic peak at 2868 cm^−1^ is attributed to cellulose's aliphatic saturated C–H stretching vibration. Furthermore, the appearance of a peak at 1430 cm^−1^ reveals information about the crystalline structure of cellulose, specifically related to the CH_2_ bending vibration. Prior research [39] reported that the intensity of this peak may indicate the degree of crystallinity within the cellulose substance. Peaks in the spectra of nopal powder are visible within 1430 cm^−1^ to 1630 cm^−1^ spectral area, and they serve as accurate determinants for the presence of lignin while being connected to C–C vibrations [40]. Also, the bands in range from 1250 cm^−1^ to 1400 cm^−1^ display C–O stretching vibrations caused by aliphatic primary and secondary alcohols located in cellulose. According to other investigations [41,42], the expansive peaks at 1050 cm^−1^ may be called ether connections (C–O–C). Moreover, nopal powder displays a high peak at 2349 cm^−1^, indicating the presence of carbon dioxide molecules.

### 3.2 SEM characterization

**Fig. 5:**
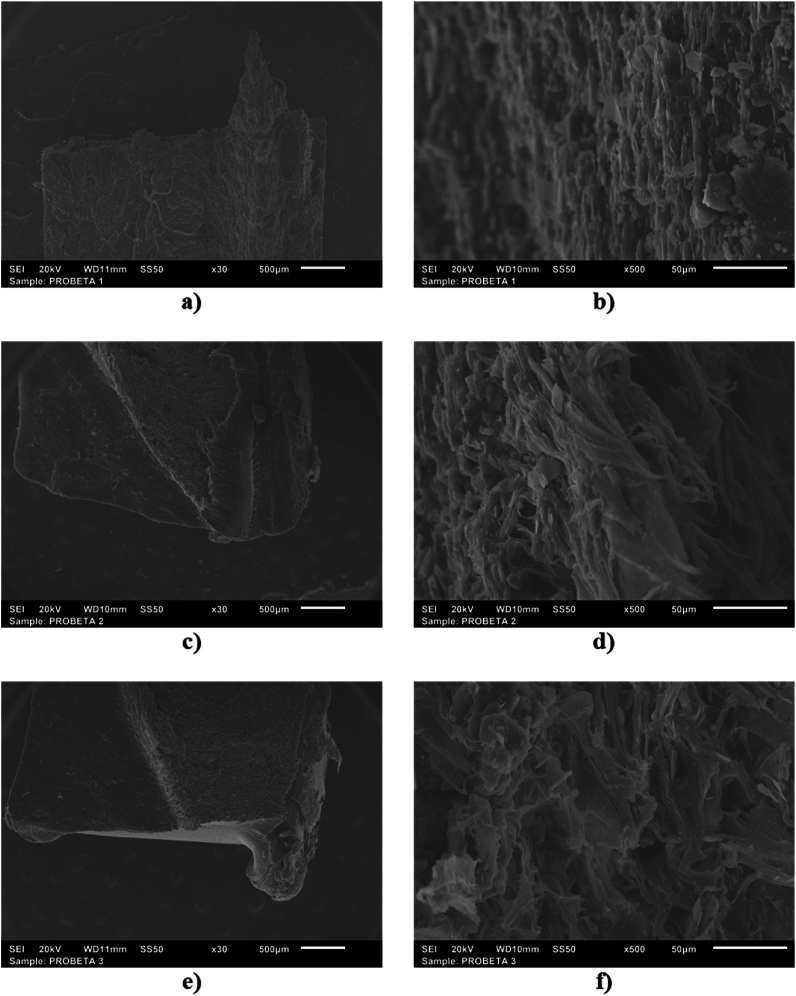
SEM images of (a,b) M1-nopal powder sample, (c–d) M2-nopal powder sample, and (e,f) M3-nopal powder sample used as triboelectric layers in the proposed TENGs.

We employ the scanning electron microscope (SEM) to examine the surface morphology of the three nopal types used as triboelectric layers. Fig. 5(a–f) illustrates the SEM images of three (M1, M2, and M3) nopal powder samples, respectively. The SEM images show the non-uniform distribution of the surfaces of the three nopal types, which can increase the roughness and surface contact area of the triboelectric layers. This increase in the surface contact area can improve the tribological performance of the nanogenerators.

### 3.3 Electrical performance characterization

**Fig. 6:**
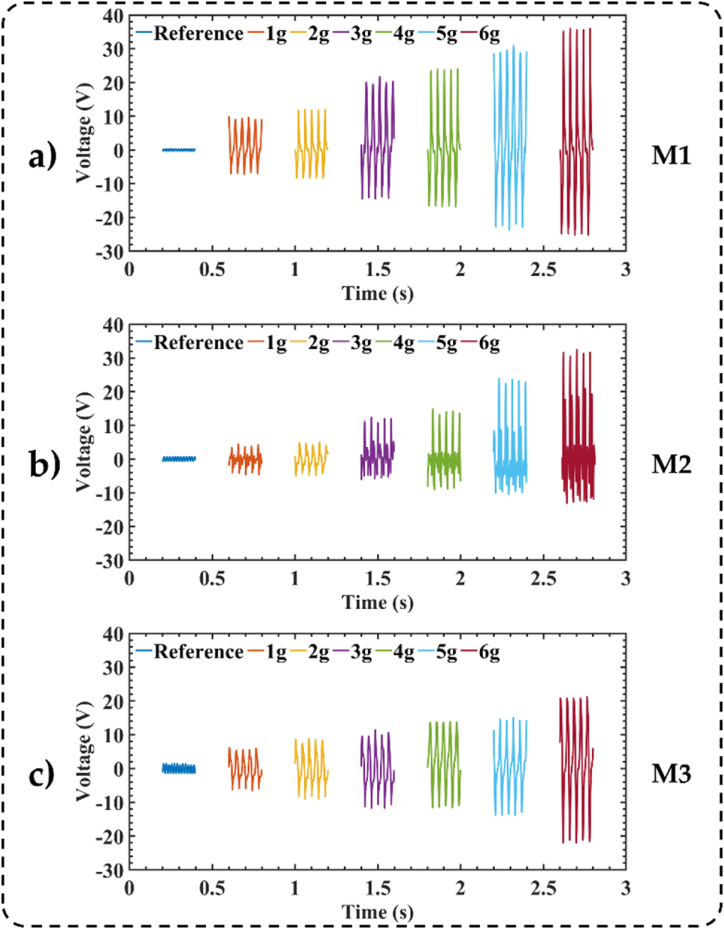
Measurements of the peak-to-peak open circuit voltage of the three nopal powder-based TENGs under six different accelerations. (a) (M1) fresh nopal powder-based TENG, (b) (M2) dehydrated nopal powder-based TENG, and (c) (M3) old nopal powder-based TENG.

We assessed the output performance of three TENGs based on different nopal types (M1, M2, and M3) at 25 Hz using six different accelerations (i.e., 1g, 2g, 3g, 4g, 5g, and 6g). Fig. 6 depicts the peak-to-peak open circuit voltage (*V*_*p-p*_) of these TENGs. For the TENG based on fresh nopal (M1) under accelerations of 1g, 2g, 3g, 4g, 5g, and 6g, we measured maximum *V*_*p-p*_ of 9.0 V, 10.00 V, 18.40 V, 23.80 V, 34.40 V, y 45.60 V, respectively. Video S1 of supplementary material depicts the *V*_*p-p*_ of the M1-based TENG.

**Fig. 7:**
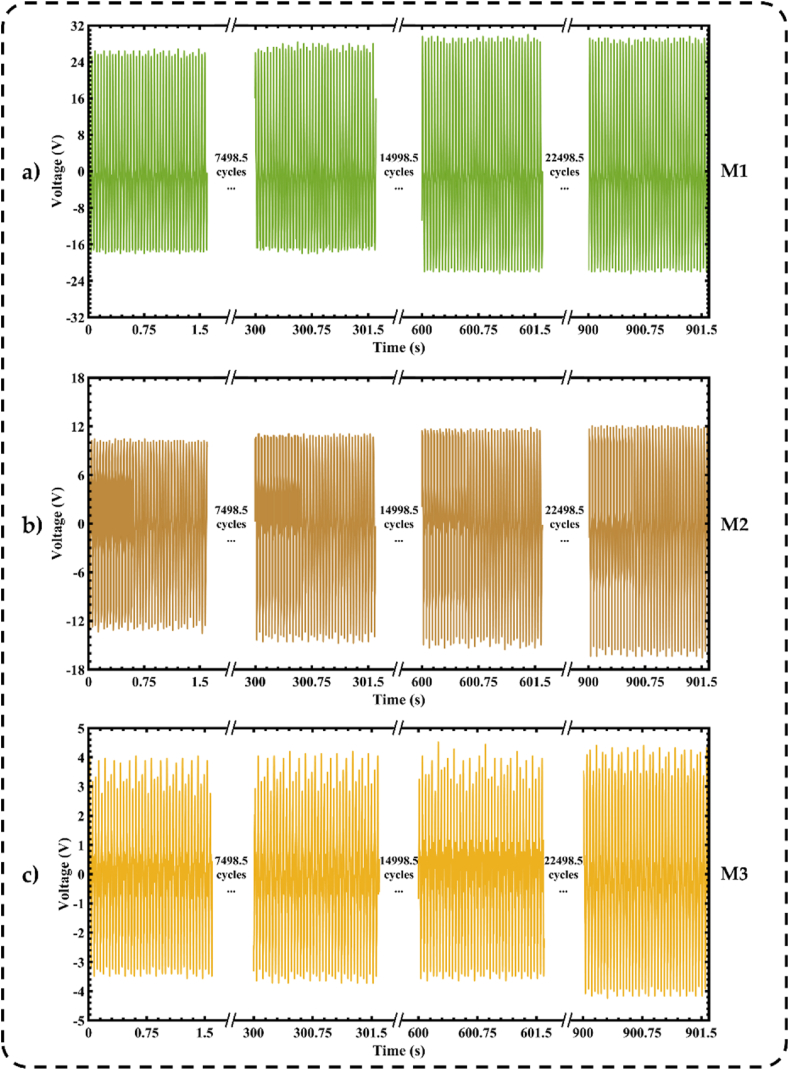
Results of the stability test of the peak-and-peak open circuit output voltage of three triboelectric nanogenerators using different nopal powder types: (a) M1-based nanogenerator, (b) M2-based nanogenerator, and (c) M3-based nanogenerator.

To study the stability of the output performance of the three nanogenerators, we measure their *V*_*p-p*_ under acceleration of 3g at 25 Hz during 22500 operating cycles, as shown in Fig. 7. This study evaluates the three TENGs considering the different nopal powder conditions: M1-based TENG, M2-based TENG, and M3-based TENG. The experiment results depict the variation of the *V*_*p-p*_ of each TENG as a function of time. The M1-based TENG has the best output voltage stability, with voltage values between 44.8 V and 52.4 V, while the M2-based nanogenerator reports a minor variation between 7.6 V and 8.6 V. Furthermore, the M3-based nanogenerator registers constant *V*_*p-p*_ from 24.0 V to 28.6 V. According to these results, the fresh nopal powder-based nanogenerator has a very stable behavior of its output voltage with a slowly increasing trend. This increase may be attributed to the contact electrification effect between both triboelectric materials of the nanogenerator.

**Fig. 8:**
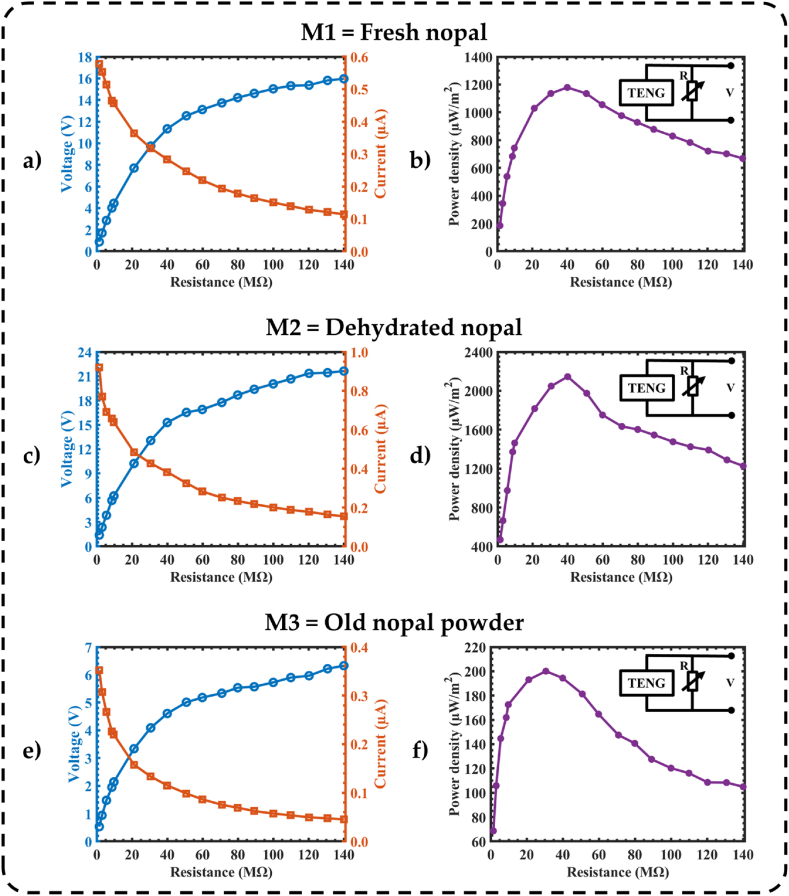
Measurements of the output performance of the three triboelectric nanogenerators using different nopal powder types. (a) The output voltage, current, and (b) output power density of the M1-based TENG as a function of load resistance. (c) The output voltage, current, and (d) output power density of M2-based triboelectric nanogenerator under different load resistances. (e) The output voltage, current, and (f) output power density of M3-based TENG at different load resistances.

The three nopal powder-based nanogenerators were subjected to an acceleration of 3g at a frequency of 25 Hz, while their output power densities were measured as a function of load resistance (*R*_*L*_). The load resistances have values from 1.5 MΩ to 140 MΩ, and an electrical connection is established between the two electrodes of the nanogenerators. In the range of 1.5 MΩ–140 MΩ, the output voltage of the three nanogenerators increases, while the output current shows an inverse trend, decreasing when the load resistance increases. Based on the test results, the M1-based TENG generates a power density of 1179.02 μWm^-2^, an output voltage of 11.33 V, and a current of 0.28 μA under a load resistance of 39.97 MΩ (Fig. 8(a and b)). This current is calculated using Ohm's law. On the other hand, the M2-based nanogenerator has a power density of 2145.24 μWm^-2^, an output voltage of 15.28 V, and a current of 0.38 μA with a load resistance of 39.97 MΩ (Fig. 8(c–d)). Finally, the M3-based nanogenerator registers a power density of 200.07 μWm^-2^, an output voltage of 4.08 V, and a current of 0.13 μA with a load resistance of 30.61 MΩ (Fig. 8(e and f)).

**Fig. 9:**
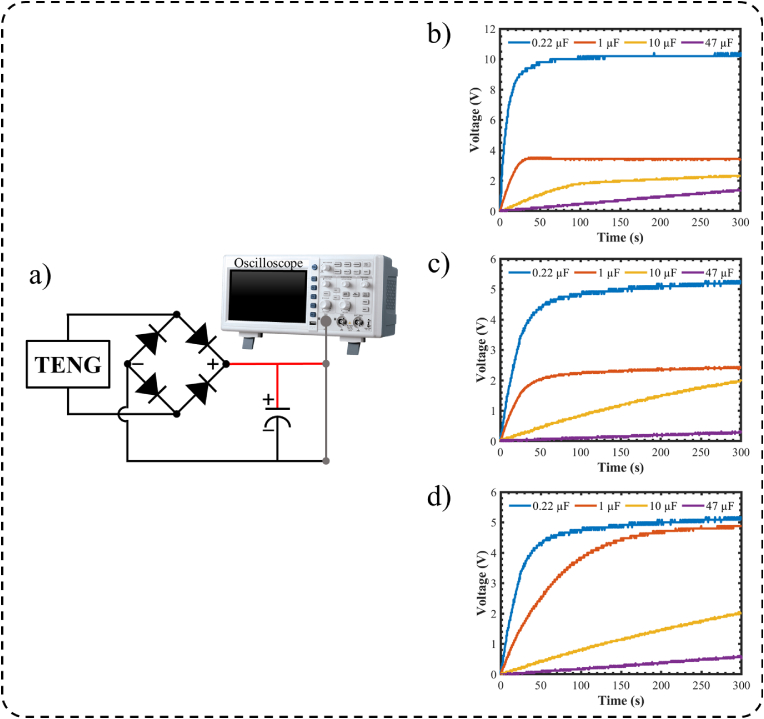
Connection diagram of the electrolytic capacitors to the (a) proposed TENG and voltage stored in capacitors of 0.22 μF, 1 μF, 10 μF, and 47 μF using (b) M1-based TENG, (c) M2-based TENG, and (d) M3-based TENG.

The three nopal powder-based nanogenerators can generate AC voltage signals with stable behavior. By using a rectifier circuit, these AC signals can be rectified to DC signals and stored in a capacitor. Thus, we connected the output of each nanogenerator to a commercial diode bridge and used electrolytic capacitors to store the DC signals of the diode bridge (Fig. 9(a)). Subsequently, the nanogenerators were placed on a shaker oscillating with an acceleration of 3 g at 25 Hz. The rectified signal of each nanogenerator was employed to charge four different capacitors. Fig. 9(b–d) illustrates the charging curves of the four capacitors (0.22 μF, 1 μF, 10 μF, and 47 μF) using the output voltage of the three triboelectric nanogenerators. Thus, the nopal powder-based TENGs can generate AC signals that can be rectified and stored in a capacitor to power small electronic devices.

### 3.4 Applications

**Fig. 10:**
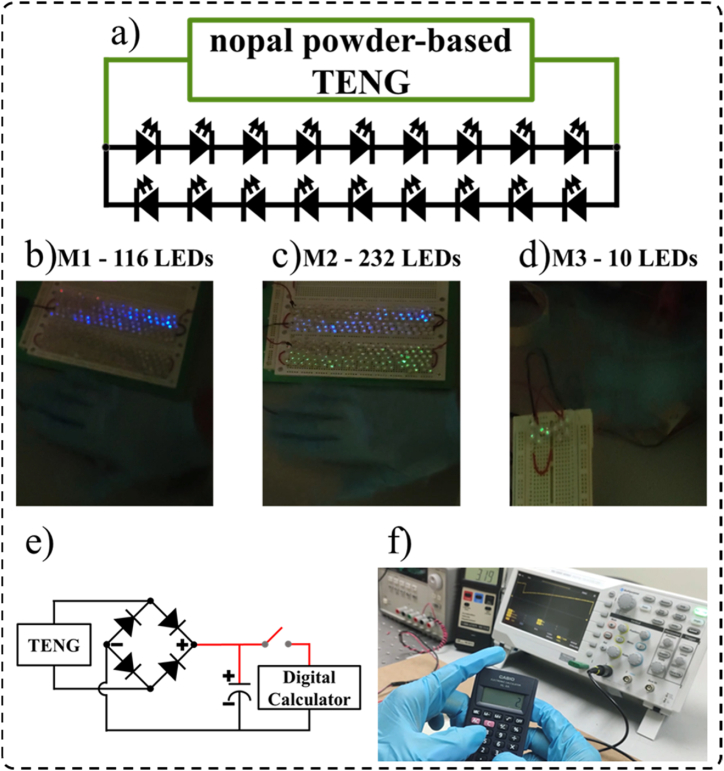
Applications of nopal powder-based TENGs. (a) The electrical circuit of the nopal powder-based TENGs is connected to commercial LEDs. (b) 166 blue LEDs lighted using the M1-based TENG. (c) 116 green and 116 blue LEDs lighted using the M2-based TENG. (d) 10 green LEDs lighted using the M3-based TENG. (e) The electrical circuit of the M1-based TENG with a diode bridge, capacitor of 10 μF, and digital calculator. (f) The energy scavenged from M1-based TENG is used to power a digital calculator. (For interpretation of the references to colour in this figure legend, the reader is referred to the Web version of this article.)

Videos S2, S3, and S4 of supplementary material depict 10 green LEDs, 116 blue LEDs, and 116 green and 116 blue LEDs lighted using the M3-based TENG, M1-based TENG, and M2-based TENG, respectively (Fig. 10(a–d)). In addition, the output of the M1-based TENG is connected to a commercial diode bridge to convert its AC output voltages into DC output voltages (Fig. 10(e)). These DC output voltages are stored using a capacitor of 10 μF. Thus, the voltage stored in the capacitor is used to power a digital calculator for approximately 5 s, as shown in video S5 of supplementary material and Fig. 10(f). By considering these applications, the proposed TENGs can harvest renewable vibration energy to power electronic devices.

**Fig. 11:**
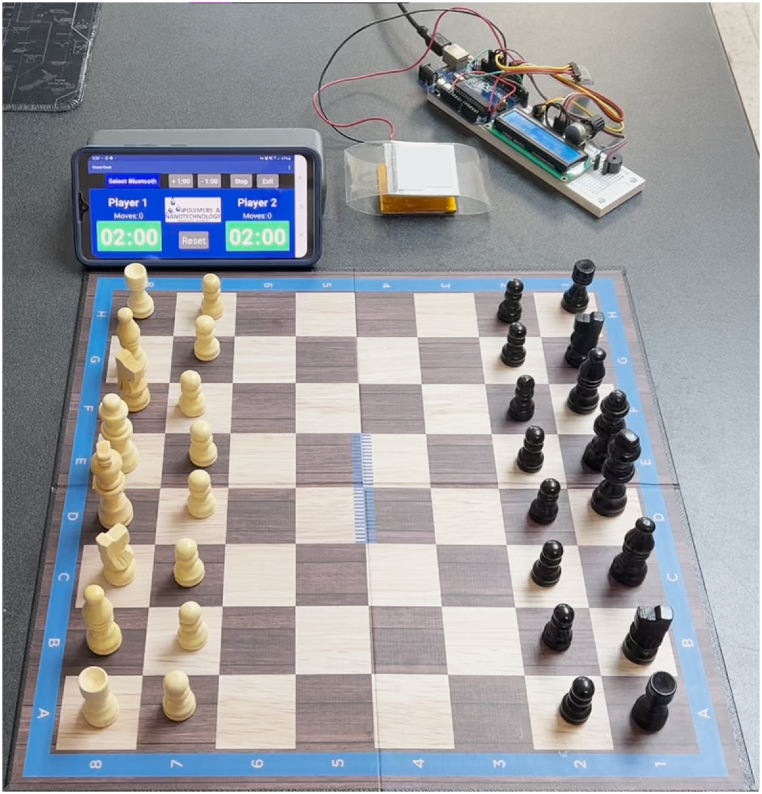
Fresh nopal powder-based TENG is used as a button for the chess clock.

Finally, our triboelectric nanogenerator is employed to build a chess clock, including an Arduino circuit board and mobile phone app (Fig. 11). This application demonstrates the adaptability of the proposed nanogenerator in a variety of practical applications. This nanogenerator can convert mechanical motion into electrical signal. Thus, our nanogenerator can operate as a mechanical button for a chess clock, integrated with an Arduino circuit board and a mobile device. The nanogenerator generates an electrical signal when a force is applied with the hand on the M1-based TENG, which is read on an ARDUINO one. Each time the nanogenerator is pressed, a value is recorded and stored to count the number of times it has been pressed. For each mechanical interaction with the nanogenerator, data are sent through a Bluetooth module to a mobile phone. The mobile device reads the data transmitted to start the countdown of a timer clock used to play chess. Furthermore, the proposed TENG can generate electrical energy from vibrational energy sources using sustainable and eco-friendly materials. Also, this nanogenerator can perform as a self-powered sensor to measure the mechanical motion of the surroundings. Video 6 of the supplementary material illustrates the chess clock composed of the M1-based TENG, an Arduino circuit board, and a mobile device.

### 3.5 Advantages and limitations

Our eco-friendly and sustainable triboelectric nanogenerator can be manufactured at low cost using waste and recycled materials with a portable and compact design that allows high output performance and stable output voltage under different vibration accelerations of the surroundings. In addition, the TENG design can facilitate its transportation and collocation on different non-homogeneous surfaces of vibration or motion sources to convert their kinetic energy into electrical energy. In addition, this nanogenerator could function as a self-powered sensor to detect environmental vibrations or motion. The nanogenerator's output performance can be improved under higher accelerations and vibration frequencies. Also, this output performance can be increased by optimizing the surface area, thickness, and separation distance of the triboelectric layers.

On the other hand, the integration of triboelectric nanogenerators with energy storage devices is an important challenge. To solve this problem, energy management techniques for the TENGs can be considered by including high-efficiency and low-consumption electrical interfaces [43,44]. Another challenge is the selection of materials that increase the durability of the nanogenerators. The wear of the triboelectric layers due to their mechanical interactions or friction can decrease their durability. To reduce this wear, the nopal powder must be firmly adhered to the copper electrode to provide a more uniform surface layer and extend the lifetime of the triboelectric film. Furthermore, more research is needed to determine the lifetime of triboelectric layers from natural polymers, considering their size and thickness, as well as the humidity of the surroundings.

The structure of the nanogenerator is lightweight, small, and portable, which is suitable for green energy harvesting from various vibration sources. For instance, our nanogenerator can be transported and collocated on different surfaces subjected to random vibrations. Additionally, the use of recycled or organic materials contributes to a circular economy by reducing waste and environmental harm. As a result, nopal powder could stick to surfaces made of recyclable materials, utilizing glue coatings to develop sustainable and eco-friendly TENGs. Table 1 shows the comparison of the output performance of various TENGs fabricated with natural polymers.

**Table 1:** Comparison of output performance and parameters of various triboelectric nanogenerators based on natural and synthetic polymers.

Triboelectric layer from natural polymer	Opposite triboelectric film	Triboelectriclayer area	Output power density	Open circuit voltage	Advantages	Reference
**Fish fin**	Polytetrafluoroethylene	*---*	*---*	130 V	Biodegradable and flexible material	[10]
**Celery cabbage**	Bread	48 cm^2^	1 mWm^−2^	15 V	Plastic-free, eco-friendly, and stable voltage	[30]
**Chicken skin**	Kapton	3 × 3 cm^2^	2000 mWm^−2^ at *R*_*L*_ of 20 MΩ	123 V	Biocompatible, eco-friendly, and high performance	[31]
**Pistachio**	PTFE	4.5 × 4.5 cm^2^	4161.4 mWm^−2^ at *R*_*L*_ of 3 MΩ	700 V	Low-cost fabrication and eco-friendly materials	[32]
**Rumex *vesicarius* leaves powder**	Poly (ethylene terephthalate)(PET)/Polytetrafluoroethylene- ethylene (PTFE) film	5 × 5 cm^2^	1.894 mWm^−2^ RL of 20 MΩ	3.86 V	Simple design, low-cost manufacturing, and stable output performance	[33]
**Sunflower husk powder**	PET	5 × 5 cm^2^	480 mWm^−2^ at *R*_*L*_ of 3 MΩ	488 V	Low-cost and eco-friendly material	[34]
**Peanut shell powder**	PET	4.5 × 4.5 cm^2^	577 mWm^−2^ at *R*_*L*_ of 5 MΩ	390 V	Bio-waste triboelectric layer	[45]
**Diatom frustule- chitosan**	FEP	3 × 4 cm^2^	15.7 mWm^−2^ at *R*_*L*_ of 5MΩ	150 V	Simple structure and low-cost manufacturing	[46]
**Dehydrated nopal powder**	Polyamide film	5.22 × 5.22 cm^2^	0.556 mWm^−2^ at *R*_*L*_ of 76.89 MΩ^a^ 2.309 mWm^−2^ at *R*_*L*_ of 76.89 MΩ^b^	16.4 V 38 V	Portable and compact structure, effective-cost manufacturing process, and stable performance	[47]
**Fresh nopal powder** **Dehydrated nopal powder** **Old nopal powder**	Polyamide film	5.22 × 5.22 cm^2^	1.179 mWm^−2^ at *R*_*L*_ of 39.97 MΩ^c^ 2.145 mWm^−2^ at *R*_*L*_ of 39.97 MΩ^d^ 0.20 mWm^−2^ at *R*_*L*_ of 30.61 MΩ^e^	11.33 V 15.28 V 4.08 V	Waste and recycled materials, portable design to use on non-homogeneous surfaces of vibration sources, high output performance, stable output voltage, and low-cost fabrication	This work

*--* Data not available.

a Test using the shaker.

b Test by applying the hand force.

c M1-based TENG test using the shaker.

d M2-based TENG test using the shaker.

e M3-based TENG test using the shaker.

## 4 Conclusions

Three nopal powder types were assessed as triboelectric layers of eco-friendly and sustainable TENGs to scavenge renewable vibration energy and power electronic devices. In addition, the design of TENGs included a polyimide triboelectric layer and a recycled PET sheet. The design of the nanogenerators allowed compact and portable structures developed using waste and recycled materials and a low-cost fabrication process. In addition, the nanogenerators presented a simple transduction mechanism and easy signal processing to achieve high output performance and stable output voltage for 22500 operating cycles. The nanogenerators were employed to power commercial LEDs and an electronic calculator. Also, the fresh nopal powder-based TENG was used to build a chess clock for smart motion sensing. The nanogenerators composed of eco-friendly and recycled materials can contribute to the familiar economy, reusing the waste materials to convert the kinetic energy from the surroundings into electrical energy. These nanogenerators could be placed on different vibration sources to power smart devices.

Future research works will include the study of the wear of the triboelectric layers and the effect of the variations of the environmental relative humidity and temperature on the performance of the nanogenerators.

## CRediT authorship contribution statement

**Enrique Delgado-Alvarado:** Writing – original draft, Methodology, Investigation. **Jaime Martínez-Castillo:** Visualization, Supervision. **Enrique A. Morales-González:** Supervision, Conceptualization. **José Amir González-Calderón:** Resources, Formal analysis. **Edgar F. Armendáriz- Alonso:** Validation, Investigation. **Gustavo M. Rodríguez-Liñán:** Validation, Formal analysis. **Ricardo López-Esparza:** Methodology, Investigation. **José Hernández-Hernández:** Visualization, Resources. **Ernesto A. Elvira-Hernández:** Investigation, Formal analysis. **Agustín L. Herrera-May:** Writing – review & editing, Investigation.

## Declaration of competing interest

The authors declare the following financial interests/personal relationships which may be considered as potential competing interests:Agustin L. Herrera-May reports financial support and administrative support were provided by Veracruzana University.
