# Supplementary material for: Eco-friendly, compact, and cost-efficient triboelectric nanogenerator for renewable energy harvesting and smart motion sensing
Source: Heliyon. 2024 Mar 25;10(7):e28482. doi: 10.1016/j.heliyon.2024.e28482 (PMC11004540; doi:10.1016/j.heliyon.2024.e28482)
Supplement: Multimedia component 7 [file mmc7.docx]

**Supplementary Information**

**Eco-friendly, compact, and cost-efficient triboelectric nanogenerator for renewable energy harvesting and smart motion sensing**

Enrique Delgado-Alvarado^1^, Jaime Martínez-Castillo^1^, Enrique A. Morales-González^1^, José Amir Gonzalez-Calderón^2^, Edgar F. Armendáriz- Alonso^3^, Gustavo M. Rodríguez-Liñán^4^, Ricardo López-Esparza^5^, José Hernández-Hernández^6,7^, Ernesto Elvira-Hernández^6^ and Agustín L. Herrera-May^1,7*^

^1^ Micro and Nanotechnology Research Center; Universidad Veracruzana, Boca del Río 94294, Veracruz, Mexico

^2^ Cátedras CONAHCYT-Instituto de Física, Universidad Autónoma de San Luis Potosí, San Luis Potosí 78290, San Luis Potosí, Mexico

^3^ Doctorado Institutcional en Ingenieria y Ciencia de Materiales, Universidad Autónoma de San Luis Potosí 78210, San Luis Potosí, Mexico

^4^ Investigadores por Mexico, Centro de Geociencias, Universidad Nacional Autónoma de Mexico, Juriquilla 76230, Querétaro, Mexico

^5^ Departamento de Física, Universidad de Sonora, Hermosillo 83000, Sonora, Mexico

^6^ Facultad de Ingeniería Mecánica y Ciencias Navales, Universidad Veracruzana, Boca del Río 94294, Veracruz, Mexico

^7^ Maestría en Ingeniería Aplicada, Facultad de Ingeniería de la Construcción y el Hábitat, Universidad Veracruzana , Boca del Río 94294, Veracruz, Mexico

*Corresponding author: *Email address*: leherrera@uv.mx (A.L. Herrera-May)

**Supplementary figure S1**

Figure S1 illustrates the three nopal powder samples (M1, M2, and M3) used as triboelectric layers of the proposed TENG.


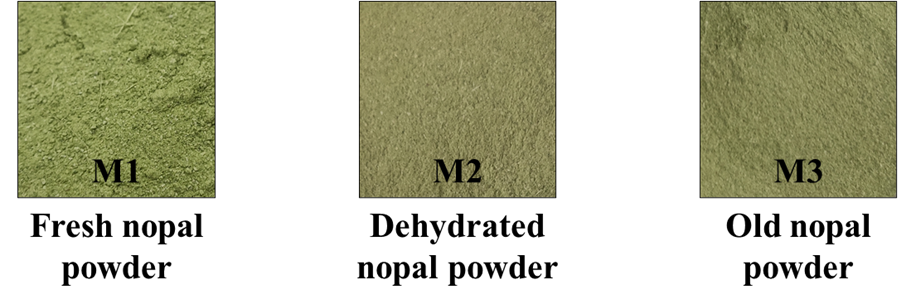


**Fig. S1**. Photography of three nopal powder samples (M1, M2, and M3) used as triboelectric materials for the proposed TENGs.

**Supplementary video S1**

Video S1 shows the output voltage of the (M1) fresh nopal powder-based TENG under a force applied on its exterior surface. The TENG performance is tested using the force applied from two fingers, three fingers, and a palm.

**Supplementary video S2**

Video S2 depicts the (M3) old nopal powder-based TENG lighting 10 green LEDs when a hand force is applied to the TENG surface.

**Supplementary video S3**

Video S3 illustrates the (M1) fresh nopal powder-based TENG lighting 116 blue LEDs when a hand force is applied to the TENG surface.

**Supplementary video S4**

Video S4 shows the (M2) dehydrated nopal powder-based TENG lighting 116 green and 116 blue LEDs when a hand force is applied to the TENG surface.

**Supplementary video S5**

Video S5 depicts the (M1) fresh nopal powder-based TENG powering a digital calculator for 3 seconds.

**Supplementary video S6**

Video S6 shows the (M1) fresh nopal powder-based TENG for motion sensing of the hands of chess players. Fig. S2 illustrates the chess clock using this TENG, an ARDUINO one, and a mobile phone app.


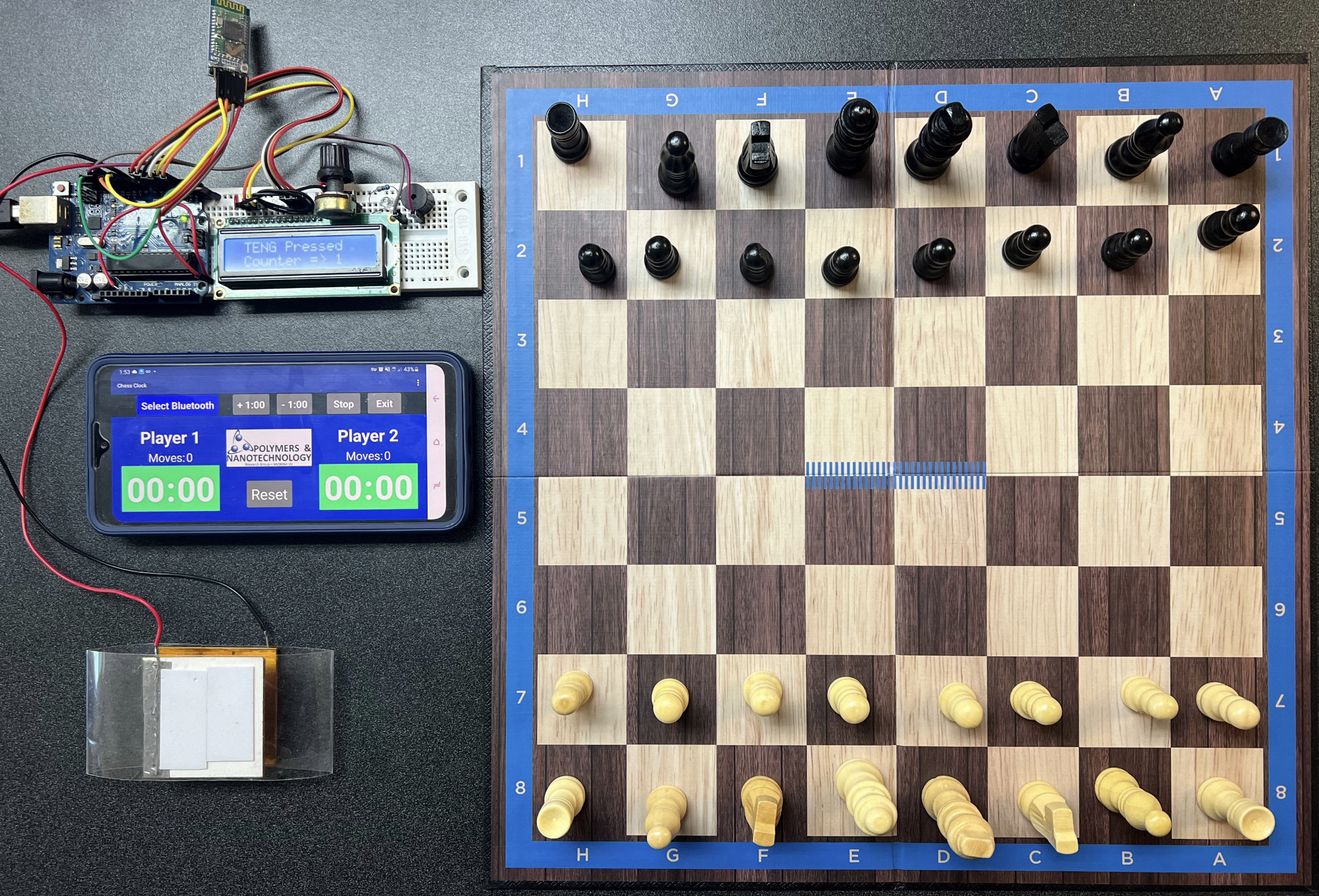


**Fig. S2**. Chess clock activated by the fresh nopal powder-based TENG.
